# Supplementary material for: A realist review of health passports for Autistic adults
Source: PLoS One. 2023 Sep 8;18(9):e0279214. doi: 10.1371/journal.pone.0279214 (PMC10490970; doi:10.1371/journal.pone.0279214)
Supplement: Attachment — Submitted filename: Response to Reviewers.docx [file pone.0279214.s002.docx]

Dear Doctor Mahmoud,

***Re:  A realist review of health passports for Autistic adults***

***PONE-D-22-32749 - [EMID:9903eae1a784495f]***

Thank you for providing such a positive and supportive response to this article. Below I have outlined the changes made in response to the reviewers’ comments and journal requirements. The reviewer’s comments are reported, with our response indented and italicised for clarity. Within the manuscript, I have used yellow highlighting to indicate areas that have been revised.

**Reviewer #1:**

1. Title: Could be more concise, or restructured to draw the immediate focus to the main purpose of the research (e.g. open with “A realist review of health passports for autistic adults” rather than the summary of the findings)

*Thank you for drawing this to our attention, on reflection, we have updated the title of the article to: A realist review of health passports for Autistic adults.*

2. BACKGROUND: As the article and abstract open with “Autism is a normal part of cognitive diversity”, it may be helpful to use the Background to signpost towards literature on current understandings of neurodiversity for readers who may be less familiar and want to know more about this, as many readers from a medical background may be more familiar with the pathology paradigm.

*Thank you for this suggestion. We have added more references to the background section to ensure readers are signposted to neurodiversity-affirming material. These can be found lines 48-52.*

3. “The case for a realist evaluation”: It may be useful here to reference realist theory and set the scene on what a realist review entails; the Context, Mechanisms and Outcomes framework is introduced without much context.

*We apologise for not setting out the theoretical framework earlier in the article. We agree that this was an omission, and the section “the case for a realist evaluation” has been amended to fix this. This section (lines 118-133) now includes more on realist theory and an explanation of the individual components within a realist review.*

4. METHOD: Exclusion criteria: The rationale for focusing only on adults and for omitting studies published before 2010 was not immediately apparent.

*To address this omission, additional content has been added to the study selection and inclusion/exclusion section (Lines: 183-185).*

5. Table 2: Where the population in an included study is expressed as “people with intellectual disability” or similar, was the study included despite differences between autism and ID because the authors of original articles expressly included autism under their definition of ID or is this an assumption made by the reviewers? It seems there is some inconsistency about which populations are included in the review; perhaps it would be helpful to indicate more clearly whether the target population were sometimes included in broader samples.

*Thank you for pointing out that this could be clearer for future readers. The following has been added to the paragraph above table 2 to add clarity: “Some of the tools described were only for Autistic patients (n=6); others were not Autism-specific but they reported on health passport use by Autistic people (n=7)*, as well as people with Intellectual Disabilities (ID), or Intellectual and Developmental Disabilities (IDD), referred to as a Learning Disability within a UK* ***C****ontext.”*

**As an example, the Blair paper (2013), says relatively little about how their tool related to autistic people but had a case study with a single autistic patient on page 61.*

*In addition, the following has been added to the inclusion criteria: “Phenomenon: HPs that were either (i) exclusively for the use of Autistic people, or (ii) were for a broader population of patients and reported on their use with Autistic patients”. (Lines 191-193).*

6. Context: The sentence “Within realist evaluation, Context is defined as the observable social, economic, political, and cultural structures which in turn inform if Mechanisms are triggered or not.” might be worth introducing earlier in the background or methodology section to help define the concepts involved in a realist review.

*The introductory sentence quoted above has been moved to the “case for a realist evaluation” section. The two sentences introducing mechanisms and outcomes, in a similar fashion to that of context, have also been moved to this section. These can be found in lines 118-133.*

7. Where autism-related barriers are mentioned (e.g. communication, sensory differences), it may be worth emphasising that barriers for autistic people are not typically caused by autism itself but by autistic traits interacting with poorly-adapted features of services such as environments.

*Thank you for emphasising this neurodiversity affirming perspective. We completely agree with this point. Within the “context section” we have added “It is important to note here these barriers are not caused by the autistic qualities themselves, but by the interaction of these traits with poorly adapted services and environments.[8]” (Lines 321-323).*

8. Figure 2: The findings in the ‘Context’ section reference Figure 2 but do not translate directly onto the concepts in the figure, which introduces some additional information beyond what is discussed in the results that appear to be drawn from the wider context of what is known from literature in this area - some of this is expanded upon in the discussion so may be helpful for the discussion to refer to Fig.2 where relevant, and for Fig.2 to be accompanied by an explainer signposting to relevant sections of the manuscript. The resolution of Fig.2 also makes it hard to read (including in the downloadable version).

*I apologise for the poor quality of figure 2. A higher resolution copy of image 2 has been created and uploaded in the correct format. Figure 2 is now also referenced within the discussion and has been moved further down in the manuscript to address this correction.*

9. The discussion of other tools that have been developed for different populations could be moved to the background section as it provides some relevant background but seems less relevant to the discussion.

*The discussion of other tools have been moved to the background section. These are now found in lines 70-86.*

10. “Accordingly, training…” – would it be possible to present some examples of how a neurodivergent-affirming/social model approach would look different and how this could be attended to in design of interventions and research.

*Thank you for suggesting the inclusion of examples of neurodivergent-affirming practices. Examples and references have been added and can be found – lines 556-561.*

11. The review highlights how coproduced approaches and attention to theories of change appeared to lead to more positive outcomes, but state overall that AHPs are ‘unwarranted’ and ‘do not improve the accessibility of healthcare’; would it be more appropriate to suggest that future research and decision-making requires refinement as well as more attention to the CMOs identified here? This could encourage future researchers and clinical decision-makers not to abandon these tools but to improve their usage and research, which would be more consistent with the recommendations made by the researchers in the section on future directions for research.

*Thank you for this recommendation, I have added the following in “directions for future research” for better clarity:* *“There is some promise from co-produced approaches, however, we would argue the co-production process likely contains important mechanisms. HPs may not achieve the same outcomes independent of co-productive practices (White et al. 2017).” (See lines 668-671). As an example, another study we were involved in involved the development of a youth drug prevention intervention, and research processes, including the keeping of a research diary, were retained in the future intervention as they were considered an important mechanism.*

*Also, within “directions for future” research, there has been an additional sentence added: “Additionally unanticipated outcomes should be considered in evaluations.” (Line 649).*

*There have also been amendments made to the “unwarranted” paragraph in the discussion - referenced in the above recommendation. These changes can be found in lines: 524-527.*

*Within the conclusion section of the abstract, the following has also been added: “Using an AHP tool alone in a healthcare Context that does not meet Autistic needs, without the inclusion of the local Autistic community developing the tool, and a wider intervention to reduce known barriers to health inequality, may mean that AHPs do not trigger any Mechanisms, and thus cannot affect Outcomes.” (Lines 38-42.)*

12. The clinical implications section appears less developed than other sections. Are there any tangible actions the authors can suggest to improve implementation of healthcare for autistic people based on the review findings? For example, Nicolaidis’s study suggested that the CBPR approach can potentially have some positive impacts when there is a clear theory of change, understanding of context and includes other supporting elements, and may benefit from further exploration.

Are there any other approaches to addressing healthcare access and disparities for autistic adults that may work better or which still need evaluating, or are AHPs the best evidence we have at this time?

What can clinicians/healthcare managers learn from the findings of this study that could improve the application of AHPs or similar tools they may be using in their practice (e.g. professionals need to have an awareness and understanding of their purpose and motivation/confidence to use them)?

*I agree this section requires some strengthening – thank you for noting these questions as a starting point. The “Clinical Implications: supporting Autistic patients” section has been added to substantially considering this point. New information can be found between lines 588-615.*

13. Conclusions: “Researchers must take account of the barriers inherent in the intervention Context” – it may be useful to point out this can include barriers in the research design which may marginalise some autistic people from being involved in research into new interventions. Efforts should be made to reduce these as far as possible so samples are representative of the people for whom the interventions are designed and tested which will improve the applicability of findings to real life implementation.

*Thank you for highlighting this need. We have added the following to the conclusion section “We are aware that Autism research currently tends to attract people from more privileged backgrounds. Reasonable adjustments are needed to ensure research conducted is accessible for Autistic individuals who wish to be involved in health intervention evaluation and development.[77]” (Lines 685-688).*

**Reviewer #2:**

1. The only missing parts in your design were predetermined study types.

*I have added the following to the inclusion/exclusion criteria section: “We did not exclude on the basis of research design.” (Lines 183-184.)*

2. Limiting included studies to the English language and to pre-2010 publications might have caused some selection bias. However, this does not affect the importance of this review.

*Thank you for highlighting the potential selection bias in this study, I have made an addition to the limitations section (see lines 633-635) to reflect this.*

**General:**

A. **Funding Statement.**

1. We note that you have provided funding information that is not currently declared in your Funding Statement. However, funding information should not appear in the Acknowledgments section or other areas of your manuscript. We will only publish funding information present in the Funding Statement section of the online submission form. Please remove any funding-related text from the manuscript and let us know how you would like to update your Funding Statement.

*Apologies for duplication of information. The section has now been removed from the manuscript and remains in the funding statement section of the online submission form. Please could the statement be amended to the following:*

*“AG, KW, AB and EH received funding for this research from the Swansea University Accelerate Health Tech Centre. Reference: 07/09/21. RE's time was funded by this grant. The funders had no role in study design, data collection and analysis, decision to publish, or preparation of the manuscript.”*

B. **References.**

2. We note that you have referenced (ie. Bewick et al. [5]) which has currently not yet been accepted for publication. Please remove this from your References and amend this to state in the body of your manuscript: (ie “Bewick et al. [Unpublished]”) as detailed online in our guide for authors

*The paper referenced above in this correspondence could not be located within the main manuscript or references. The unpublished paper in the references section “Grant, Unpublished” has been removed and their mention within the manuscript has been changed.*

C. **Formatting requirements:**

I have updated the manuscript in line with PLOSOne formatting requirements.

D. **The following references have been added:**

[1] Leadbitter K, Buckle KL, Ellis C, & Dekker M. Autistic Self-Advocacy and the Neurodiversity Movement: Implications for Autism Early Intervention Research and Practice. *Frontiers in Psychology*, 2021; 12. DOI=10.3389/fpsyg.2021.635690

[2] Milton D. On the ontological status of autism: the ‘double empathy problem’. *Disability & Society*, 2012; 27(6), pp:883-887. Available from: <https://doi.org/10.1080/09687599.2012.710008>

[3] Dwyer P. The Neurodiversity Approach(es): What Are They and What Do They Mean for Researchers? Hum Dev. 2022; May;66(2), pp:73-92. doi: 10.1159/000523723. Available at: <https://www.ncbi.nlm.nih.gov/pmc/articles/PMC9261839/>

[38] Pawson R, & Tilley N. Realistic Evaluation. London: SAGE Publications Ltd. 1997.

[40] Trickey H, Thomson G, Grant A, Sanders J, Mann M, Murphy S, et al. A realist review of one-to-one breastfeeding peer support experiments conducted in developed country settings. Matern Child Nutr. 2017; 14:e12559. Available: <https://doi.org/10.1111/mcn.12559>

[63] Williams GL, Adams J, Bull P, Cave H, Chown N, Doherty M. et al. More than words: Supporting effective communication with autistic people in health care settings. 2022. Economic and Social Research Council. Available at: https://www.england.nhs.uk/south-east/wp-content/uploads/sites/45/2022/10/More-than-words-supporting-effective-communication-with-autistic-people-in-health-care-settings-3.pdf

[64] Doherty M, McCowan S, & Shaw SCK. Autistic SPACE: a novel framework for meeting the needs of autistic people in healthcare settings. Br J Hosp Med. 2023 Apr 2;84(4), pp:1-9. Available at: https://doi.org/10.12968/ hmed.2023.0006

[72] Burton, T. Exploring the experiences of pregnancy, birth and parenting of mothers with autism spectrum disorder. Doctoral dissertation: Staffordshire and Keele Universities. 2016.

[73] National Development Team for Inclusion. Evaluation of the Oliver McGowan Mandatory Training Trial in Learning Disability and Autism. 2022. Available at: https://www.ndti.org.uk/assets/files/OMMT-final-report.pdf

[74] Anna Freud. National Autism Trainer Programme. [Cited: 29/06/2023] 2023. Available from: https://www.annafreud.org/training/national-autism-trainer-programme/

[75] All Brains Belong VT: Neuroinclusive Healthcare & Community. [cited: 16/06/2023] 2023. Available from: <https://allbrainsbelong.org/>

[77] White J, Hawkins J, Madden K, Grant A, Er V, Angel L. et al. Adapting the ASSIST model of informal peer-led intervention delivery to the Talk to FRANK drug prevention programme in UK secondary schools (ASSIST + FRANK): intervention development, refinement and a pilot cluster randomised controlled trial. Southampton (UK): NIHR Journals Library; 2017 Oct. PMID: 29068622. DOI: 10.3310/phr05070

[78] Pellicano E, Dinsmore A, & Charman T. What should autism research focus upon? Community views and priorities from the United Kingdom. Autism. 2014 Oct;18(7), pp: 756-70. DOI: 10.1177/1362361314529627.

Again, thank you all for your supportive comments and suggestions for strengthening the paper – this has been very much appreciated.

Yours sincerely,

Rebecca Ellis & Aimee Grant

On behalf of the authors.
