# Supplementary material for: A realist review of health passports for Autistic adults
Source: PLoS One. 2023 Sep 8;18(9):e0279214. doi: 10.1371/journal.pone.0279214 (PMC10490970; doi:10.1371/journal.pone.0279214)
Supplement: Attachment — Submitted filename: Response to Reviewers.docx [file pone.0279214.s003.docx]

Dear Doctor Mahmoud,

***Re:  A realist review of health passports for Autistic adults***

***PONE-D-22-32749 - [EMID:9903eae1a784495f]***

Thank you for reviewing our work so promptly. The reviewer’s single comment is below, with our response indented and italicised for clarity. Within the manuscript, I have used yellow highlighting to indicate areas that have been revised.

1. I am satisfied the authors have addressed all the reviewers’ comments. However, one point that I noted to be inconsistent with their other arguments is on p.45 line 595: “However, the quality of this, for example if it is designed and delivered by Autistic people through a neurodiversity affirming lens, can mean that such training could reinforce existing negative stereotypes and not impact on culture change” – did the authors mean to say that if training is not delivered in this way it may reinforce negative stereotypes? Please check this wording and adjust or clarify if necessary.

*Thank you for highlighting this inconsistency. I have changed the word to make the meaning clearer in the paragraph. The paragraph has been amended to the following:*

*“However, if the quality of training surrounding this intervention is poor, it could reinforce existing negative stereotypes and not create any cultural change. Training which was, for example, designed and delivered by Autistic people, through a neurodiversity-affirming lens, could reduce inequalities.”* *p.45 (lines 595-599).*

Again, thank you all for your supportive comments and suggestions throughout the review process – this has been very much appreciated.

Yours sincerely,

Rebecca Ellis

On behalf of the authors.
